# Pro-inflammatory S100A9 contributes to retinal ganglion cell degeneration in glaucoma

**DOI:** 10.3389/fimmu.2025.1667097

**Published:** 2025-09-25

**Authors:** Zuo Wang, Yang Chen, Jinxia Wang, Wenbo Xiu, Gao Zhang, Yanping Gao, An Li, Ping Long, Bolin Deng, Chong He, Fang Lu

**Affiliations:** ^1^ Department of Clinical Laboratory, Sichuan Clinical Research Center for Cancer, Sichuan Cancer Hospital & Institute, Sichuan Cancer Center, University of Electronic Science and Technology of China, Chengdu, China; ^2^ Translational Clinical Immunology Key Laboratory of Sichuan Province, Sichuan Provincial People’s Hospital, University of Electronic Science and Technology of China, Chengdu, China; ^3^ Department of Ophthalmology, Sichuan Provincial People’s Hospital, University of Electronic Science and Technology of China, Chengdu, China; ^4^ Core Laboratory, Sichuan Provincial People’s Hospital, University of Electronic Science and Technology of China, Chengdu, China

**Keywords:** S100A9, retinal neurodegeneration, glaucoma, neuroinflammation, glial activation

## Abstract

S100A9 is a pro-inflammatory protein involved in neuroinflammation and central nervous system (CNS) neurodegenerative diseases, such as Alzheimer’s and Parkinson’s disease. Glaucoma, the leading cause of irreversible blindness, shares common pathogenic mechanisms with CNS disorders. These parallels suggest a potential role for S100A9 in glaucoma; however, its precise contribution remains unclear. In this study, we investigated the association between S100A9 and glaucoma by enrolling 121 patients with glaucoma, administering intravitreal injections of recombinant murine S100A9 (rmS100A9), and employing an elevated intraocular pressure (EIOP)–induced glaucoma mouse model. We found that circulating S100A9 levels were elevated in patients with glaucoma and positively associated with disease stage. Retinal S100A9 expression was significantly elevated and correlated with progressive retinal ganglion cell (RGC) loss in EIOP glaucoma mice. Furthermore, intravitreal injection of rmS100A9 led to direct RGC degeneration. Both enrichment analyses and experimental validation indicated that S100A9 may contribute to glaucomatous injury by promoting neuroinflammatory responses in retinal microglia and astrocyte via activation of the Toll-like receptor 4 (TLR4) pathway. These results raise the possibility that S100A9 as a potential target for future therapeutic exploration in glaucoma.

## Introduction

Glaucoma is a leading cause of irreversible vision loss worldwide, characterized by progressive degeneration of retinal ganglion cells (RGCs) and optic nerve damage (1). Globally, glaucoma currently affects an estimated 95 million individuals, with approximately 10 million experiencing vision loss in at least one eye (2). Although elevated intraocular pressure (IOP) is a major risk factor, normalization of IOP alone often fails to halt disease progression, underscoring the complex and multifactorial nature of glaucoma pathogenesis (3).

Neuroinflammation has emerged as a crucial mechanism underlying RGC damage in glaucoma (3–5), with microglia playing a pivotal role in this process (6–10). As the resident immune cells of the retina, microglia are the first responders to injury and stress, becoming activated early in the disease course (8). Once activated, microglia release a variety of pro-inflammatory cytokines and chemokines, such as tumor necrosis factor-α (TNF-α) and interleukin-1β (IL-1β), which can directly damage RGCs and exacerbate neuroinflammation (3, 8). Additionally, peripheral immunity also plays an indispensable role in the development of retinal neuroinflammation in glaucoma (11, 12). Peripheral immune mediators, such as lymphocytes and antibodies, can infiltrate the retina and might interact with microglia, further amplifying their activation (11–16). Our previous work also demonstrated the infiltration of Th1 cells into the glaucomatous retina, where their interaction with activated glia exacerbates neuronal damage (11). This crosstalk between peripheral immune mediators and retinal microglia creates a synergistic inflammatory response, thereby accelerating RGC degeneration (11). However, the precise mechanisms by which peripheral immunity participates in the pathogenesis of glaucoma remain to be fully elucidated. There are still many questions regarding which mediators are involved in retinal damage and how they exert their effects in the context of glaucomatous neuroinflammation.

The pro-inflammatory calcium-binding protein S100A9 has emerged as an important pro-inflammatory mediator in neurodegenerative diseases (17–21). It activates pattern recognition receptors such as Toll-like receptor 4 (TLR4) and the receptor for advanced glycation end products (RAGE), thereby initiating downstream inflammatory signaling cascades (17). S100A9 promotes the aggregation of amyloid-β (Aβ) into neurotoxic amyloidogenic structures, triggering an amyloid–neuroinflammatory cascade that contributes to disease progression, and enhances microglial activation, exacerbating neuronal injury in Alzheimer’s disease (AD) (17). Elevated fecal and circulation levels of S100A9 have been reported in AD and Parkinson’s disease (PD), correlating with intestinal inflammation and disease progression (18, 19). Consistent with these observations, our recent clinical data demonstrated significantly increased fecal S100A9 levels in glaucoma patients (22). Moreover, retinal transcriptome analysis indicates that *S100a9* undergoes significant changes in experimental mouse models (23). However, the precise role of circulating and retinal S100A9 in glaucoma remains unclear.

This study is the first to investigate the relationship between circulating S100A9 levels and glaucoma stage in patients. We further examined retinal S100A9 expression in an experimental glaucoma mouse model and evaluated whether intravitreal administration of recombinant murine S100A9 (rmS100A9) directly induces RGC degeneration. Through transcriptomic enrichment analyses and in experimental validation, we showed that S100A9 may exacerbate RGC loss via glial activation mediated through the TLR4 signaling cascade. These findings suggest circulating S100A9 as a candidate biomarker for glaucoma, and also raise the possibility that S100A9 might serve as a therapeutic target for this disease.

## Method

### Subjects

This study included 121 patients recruited from Sichuan Provincial people’s Hospital between January 2022 and January 2024. Ethical approval was granted by the hospital’s Institutional Review Board (Approval No. 201968), and all procedures adhered to the Declaration of Helsinki. Written informed consent was obtained from each participant for the use of clinical information. The diagnosis of glaucoma was made by experienced ophthalmologists based on comprehensive eye examinations, patient age, family history, and characteristic clinical features. Participant underwent a series of ocular tests including IOP, vertical cup-to-disc ratio (VCDR), retinal nerve fiber layer (RNFL) thickness, and visual field (VF) evaluation. IOP was measured using Goldmann applanation tonometry. RNFL, and VCDR were assessed via optical coherence tomography (OCT), and VF was evaluated using standard automated perimetry. Patients were eligible if they had a confirmed diagnosis of primary glaucoma, no history of secondary glaucoma or ocular surgery within the previous six months, and no autoimmune, inflammatory, or neurodegenerative disorders (e.g., Alzheimer’s disease or Parkinson’s disease). Glaucoma severity was classified using both the Hodapp-Parrish-Anderson (H-P-A) and the Advanced Glaucoma Intervention Study (AGIS) scoring systems. Additionally, 80 age- and sex-matched healthy individuals were recruited as health controls. Exclusion criteria for controls included a personal or family history of glaucoma, ocular pain, elevated IOP (>21 mmHg), recent ocular surgery, autoimmune, inflammatory, neurological conditions, or current use of immunomodulatory medications.

### Blood collection and ELISA

Following an overnight fast of at least 8 hours, venous blood was collected in the morning from the antecubital vein using EDTA-anticoagulant CPT tubes. In mice, blood was collected via cardiac puncture using the same type of tubes. Plasma was separated by centrifugation at 3,000 rpm for 10 minutes. Plasma concentrations of S100A9 were quantified using human- and mouse-specific ELISA kits (EH4140 and EM1620, respectively; FineTest), following the manufacturer’s instructions.

### Experimental animals and glaucoma model

Male C57BL/6 wild-type mice (10–12 weeks old) were obtained from Vital River Laboratories (Sichuan, China). All animals were handled in accordance with the protocols approved by the Association for Research in Vision and Ophthalmology Statement for the Use of Animals in Ophthalmic and Vision Research. All experimental procedures were reviewed and approved by the Animal Care and Use Committee of Sichuan Provincial People’s Hospital (No. 2019219). Mice were randomly assigned to experimental groups.

An experimental glaucoma model was established by inducing experimental ocular hypertension (EIOP) in 8- to 12-week-old mice, following previously described protocols (11, 12). Anesthesia was induced with 4% chloral hydrate (400 mg/kg, intraperitoneally), followed by euthanasia via cervical dislocation. Pupil dilation was achieved using compound tropicamide eye drops (0.5%), and topical anesthesia was administered with oxybuprocaine. To establish the experimental ocular hypertension (EIOP) model, 1.0 × 10^4^ polystyrene microbeads (1 μL) mixed with triblock copolymer hydrogel (1 μL per eye) were injected into the anterior chamber of both eyes. To minimize reflux, the glass micropipette was left in place for 2 minutes before withdrawal, and antibiotic eye ointment was applied immediately after injection. The microbeads used had a uniform diameter of 15 μm (Invitrogen). For sham controls, an equal volume of PBS (1 μL) was injected into the anterior chamber of age- and sex-matched littermate mice. After injection, animals were placed in a temperature-controlled small animal incubator for recovery. Intraocular pressure (IOP) was measured in awake mice using a TonoLab tonometer (Colonial Medical Supply), following acclimation in a plastic cone holder. Mice with IOP ≥ 25 mmHg in both eyes (≥1 measurement within 10 days for early time points; ≥3 measurements within 20 days for later time points) were considered successfully induced and included for further analysis.

### Intravitreal administration of rmS100A9

Following anesthesia with 4% chloral hydrate (400 mg/kg, intraperitoneally), rmS100A9 (R&D, Cat. No. 2065-S9) was intravitreally administered at a concentration of 40 ng/μL in a total volume of 1 μL per eye. As controls, PBS (1 μL per eye) was injected into the vitreous cavity of age- and sex-matched mice. After injection, animals were placed in a temperature-controlled small animal incubator for recovery.

### Whole-mount retina and immunostaining

Whole-mount retinas were prepared according to standard protocols. After enucleation, retinas were carefully dissected. Retinas were blocked in PBS containing 0.3% Triton X-100 and 10% donkey serum, followed by overnight incubation with primary antibodies at 4 °C. After washing, secondary antibodies were applied and incubated for 2 hours at 20 °C. Details of the primary and secondary antibodies used in this study are provided in **Supplementary Table S1**. Images were acquired using a Zeiss LSM 980 confocal microscope. ImageJ (1.54p, NIH) was used for quantitative analysis. All images and analyses were evaluated in a randomized order under blinded conditions.

### RNA extraction, qRT-PCR

Total RNA was isolated from mice retinas using TRIzol reagent (Invitrogen, USA) in accordance with the manufacturer’s instructions. Reverse transcription was performed using a commercial RT kit (Vazyme Biotech, Nanjing, China). Quantitative real-time PCR was conducted using SYBR PrimeScript reagents (Vazyme) on a CFX96 Deep Well Real-Time PCR System (Bio-Rad). Gene expression was quantified using the 2− ΔΔCT method, with β-actin used as the internal reference. The primers used for *S100a9* were as follows: forward (F1), 5′-CGACACCTTCCATCAATACT-3′; reverse (R1), 5′-TCAGCATCATACACTCCTCA-3′. The primers used for *Tlr4* were as follows: forward (F1), 5′- ATGCATGGATCAGAAACTCAGCAA -3′; reverse (R1), 5′- AAACTTCCTGGGGAAAAACTCTGG -3′.

### Enrichment analysis

RNA-seq data (GSE141725) were obtained from the Gene Expression Omnibus (GEO). Genes positively correlated with *S100a9* were subjected to Gene Ontology (GO) and Kyoto Encyclopedia of Genes and Genomes (KEGG) enrichment analysis using the enrichGO and enrichKEGG functions in the clusterProfiler package (v4.8.3) in R. Normalization used DESeq2’s median-of-ratios, with multiple testing controlled by the Benjamini–Hochberg procedure; statistical significance was defined as adjusted *p* < 0.05 and q < 0.10. Enrichment results were visualized as bar plots. Additionally, Gene Set Enrichment Analysis (GSEA) was conducted to compare the high versus low S100A9 expression groups based on all significantly differentially expressed genes (DEGs). Gene sets from the MSigDB Collections, including m5.go.bp.v2025.1.Mm.symbols.gmt (Gene Ontology Biological Process) and m2.cp.reactome.v2025.1.Mm.symbols.gmt (Reactome curated pathways), were used as reference databases. Significantly enriched pathways were defined by an adjusted *p*-value < 0.05 and an absolute normalized enrichment score (|NES|) > 1.

### Statistical analysis

All statistical analyses were performed using R software (version 4.3.3). The Shapiro–Wilk test was used to evaluate data normality, while homogeneity of variance across groups was assessed using either Bartlett’s test or the F-test, depending on the data characteristics. For comparisons between two groups, unpaired two-tailed Student’s t tests were used for normally distributed variables. If the data deviated from normality, the Mann–Whitney U test was applied. For analyses involving more than two groups, one-way analysis of variance (ANOVA) followed by Holm–Sidak’s multiple comparison test was used for parametric data. In contrast, non-parametric datasets were analyzed using the Kruskal–Wallis test followed by Dunn’s *post hoc* test. Categorical variables were analyzed using the chi-square (χ²) test. Correlation analyses were performed using Spearman’s correlation or Spearman rank-order correlation, as appropriate. To account for potential confounding factors, multivariate logistic regression analysis was performed. Odds ratios (ORs) with 95% confidence intervals (CIs) were calculated to quantify the strength of associations. Sample sizes (n) denote independent biological replicates and are specified in the relevant figure legends. All statistical tests were two-sided, with *p*-values < 0.05 considered statistically significant.

## Result

### Elevated circulating S100A9 levels in patients with glaucoma

**Figure 1:**
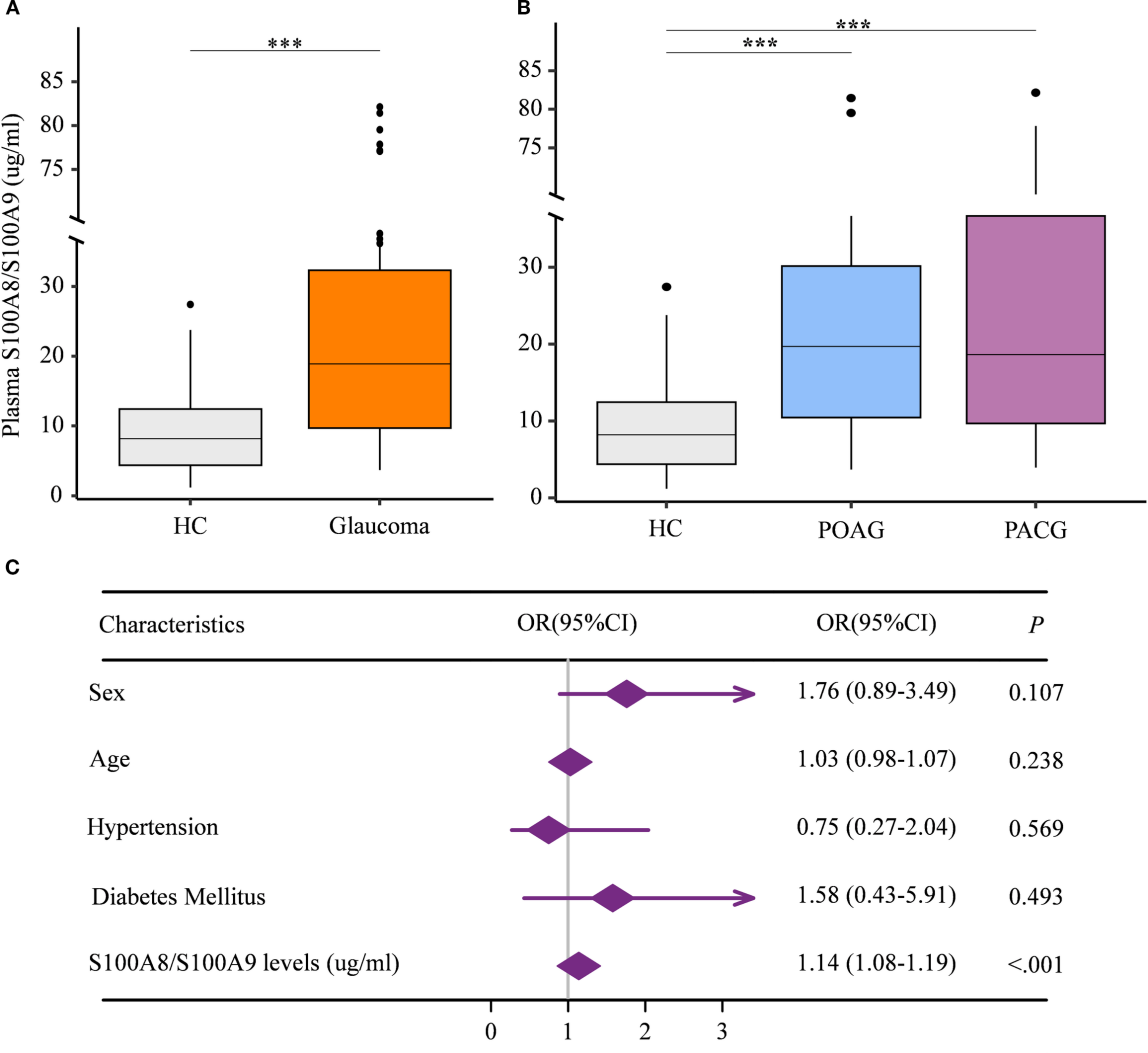
Elevated circulating S100A8/S100A9 levels in patients with glaucoma. **(A)** Circulating S100A8/S100A9 levels were compared between patients with glaucoma and healthy controls. **(B)** Circulating S100A8/S100A9 levels were further compared among glaucoma subtypes and healthy controls. **(C)** The association between circulating S100A8/S100A9 levels and glaucoma was evaluated using multivariate logistic regression. Odds ratios (ORs) were adjusted for sex (female = 1, male = 0), age (years), hypertension (yes = 1, no = 0), and diabetes mellitus (yes = 1, no = 0). Statistical analyses were conducted using **(A)** the Mann–Whitney U test for two-group comparisons, and **(B)** the Kruskal–Wallis test followed by Dunn’s multiple comparisons test for multiple-group comparisons. ***p < 0.001. Boxplots represent the median and interquartile range (IQR).

Given that the S100A8/S100A9 heterodimer is the most stable and predominant detectable form of S100A9 in peripheral circulation (24), we first assessed plasma S100A8/S100A9 levels in patients with glaucoma. A total of 121 glaucoma patients and 80 age- and gender-matched healthy controls were enrolled. Participant characteristics are summarized in **Table 1**. Plasma S100A8/S100A9 levels were measured using ELISA. Compared with healthy controls, glaucoma patients exhibited significantly elevated S100A8/S100A9 levels (**Figure 1A**). This increase was observed in both primary open-angle glaucoma (POAG) and primary angle-closure glaucoma (PACG) subtypes, with no significant difference between the two (**Figure 1B**). Interestingly, S100A8/S100A9 levels were not associated with intraocular pressure (IOP) (ρ = –0.110, *p* = 0.248). To identify potential confounding factors, multivariate logistic regression analysis was performed. After adjusting for age, sex, diabetes, and hypertension, elevated S100A8/S100A9 levels remained significantly associated with glaucoma (odds ratio [OR] = 1.14; 95% confidence interval [CI]: 1.08–1.19; *p* < 0.001) (**Figure 1C**).

**Table 1:** Demographic characteristics of glaucoma patients and healthy controls.

Variable	Healthy controls (80)	Glaucoma (121)	*P*-value
Age(year)	56.9 ± 7.6	59.2 ± 8.7	0.054
Male/female	45/35	53/68	0.085
PACG/POAG, *n*	\	76/45	\
HPA stages, *n*			
Early/Moderate/Severe	\	24/21/65	\
AGIS stages, *n*			
Early/Moderate/Severe/End-stage	\	35/27/8/40	\
Diabetes, *n* (%)	5(6.2%)	10 (8.3%)	0.596
Hypertension, *n* (%)	13 (16.2%)	18 (14.9%)	0.792

Age is presented as mean ± standard deviation (SD). Group differences in age were assessed using an unpaired two-tailed Student’s t test, and differences in gender were evaluated using the Chi-square test. *P* < 0.05 was considered statistically significant. H-P-A, Hodapp-Parrish-Anderson staging; AGIS, Advanced Glaucoma Intervention Study scoring; POAG, primary open-angle glaucoma; PACG, primary angle-closure glaucoma.

### Circulating S100A9 levels are associated with glaucoma stage in patients

**Figure 2:**
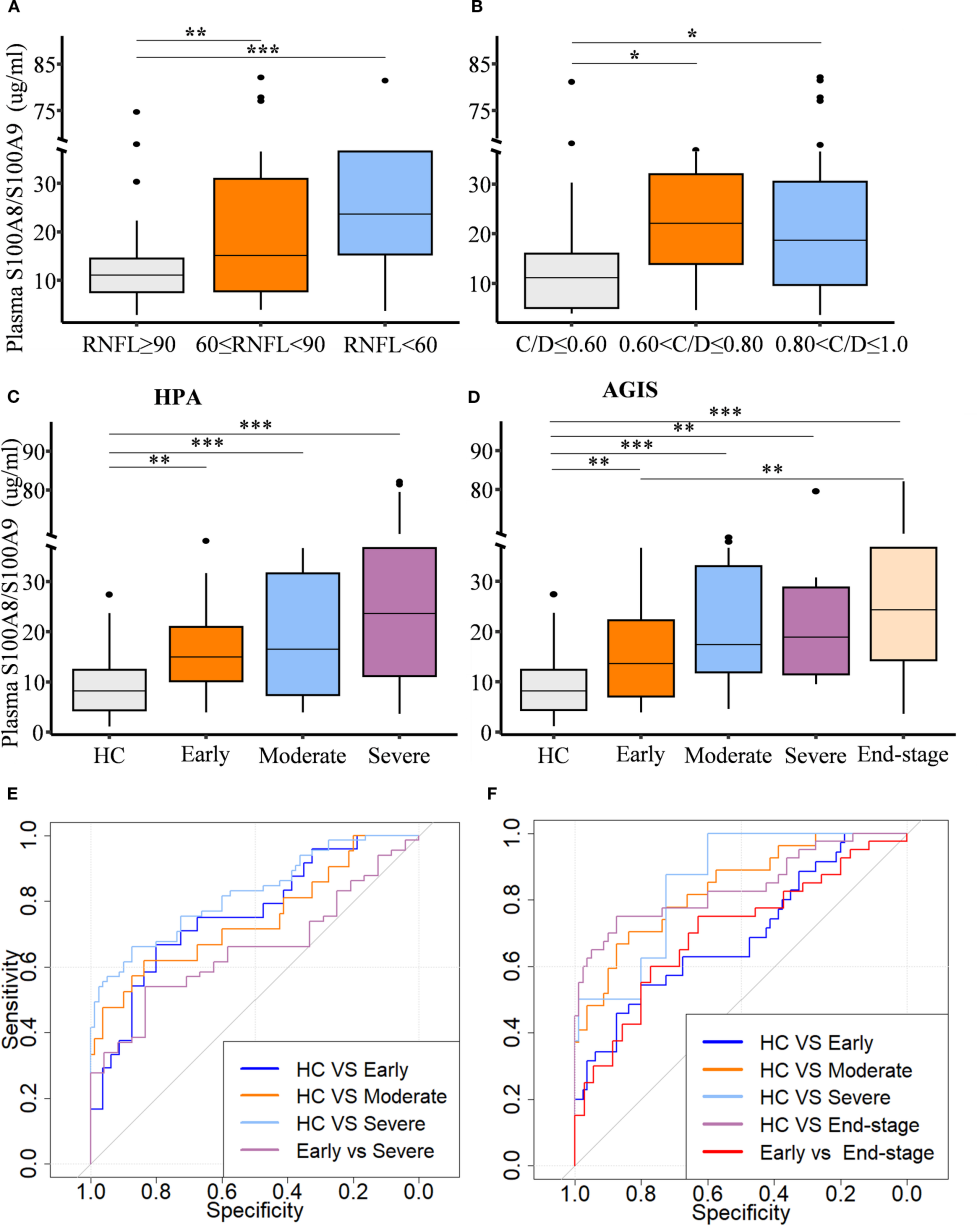
Circulating S100A8/S100A9 levels in relation to glaucoma progression. Circulating S100A8/S100A9 concentrations are shown across patient groups stratified by disease stage based on **(A)** retinal nerve fiber layer (RNFL) thickness, **(B)** cup-to-disc (C/D) ratio, **(C)** the Hodapp-Parrish-Anderson (H-P-A), and **(D)** the Advanced Glaucoma Intervention Study (AGIS) score. **(E, F)** Receiver operating characteristic (ROC) curves were generated to evaluate the discriminatory power of circulating S100A8/S100A9 levels across different HPA **(E)** and AGIS **(F)** grades. Statistical comparisons were performed using Kruskal-Wallis test followed by Dunn’s multiple comparisons test. *P < 0.05, **P < 0.01 and ***P < 0.001. Boxplots represent the median and interquartile range (IQR).

To assess the relationship between circulating S100A8/S100A9 levels and the stage of glaucomatous neurodegeneration, both structural and functional parameters were evaluated. Patients were stratified based on cup-to-disc ratio (C/D) and retinal nerve fiber layer (RNFL) thickness. S100A8/S100A9 levels were positively associated with decreasing RNFL thickness (**Figure 2A**) and increasing C/D ratios (**Figure 2B**). Glaucoma severity was further evaluated using two clinical staging systems. Based on the Hodapp–Parrish–Anderson (H-P-A) classification, patients were categorized into early, moderate, and severe stages (**Figure 2C**). According to the Advanced Glaucoma Intervention Study (AGIS) scoring system, patients were classified into early, moderate, severe, and end-stage groups (**Figure 2D**). In both systems, S100A8/S100A9 levels progressively increased with advancing disease stage (**Figures 2C, D**). Spearman rank correlation analysis confirmed significant positive associations between S100A8/S100A9 levels and glaucoma stage: H-P-A (ρ = 0.511, *p* < 0.001) and AGIS (ρ = 0.535, *p* < 0.001). Receiver operating characteristic (ROC) curve analysis demonstrated the discriminatory capacity of circulating S100A8/S100A9 levels across different disease stages. In the H-P-A classification, the area under the curve (AUC) for distinguishing healthy controls from patients with early, moderate, and severe glaucoma was 0.759, 0.745, and 0.822, respectively; the AUC for distinguishing early- from severe-stage glaucoma was 0.653 (**Figure 2E**). In the AGIS system, the AUC for differentiating healthy controls from patients with early, moderate, severe, and end-stage glaucoma were 0.692, 0.837, 0.855, and 0.840, respectively, with an AUC of 0.702 for distinguishing early- from end-stage glaucoma (**Figure 2F**).

### Progressive glaucomatous RGC degeneration is associated with elevated retinal S100A9 levels in mice

We next investigated the circulation S100A8/S100A9 and local retinal expression of S100A9 to assess its contribution to glaucomatous neurodegeneration in an experimental glaucoma mouse model.

**Figure 3:**
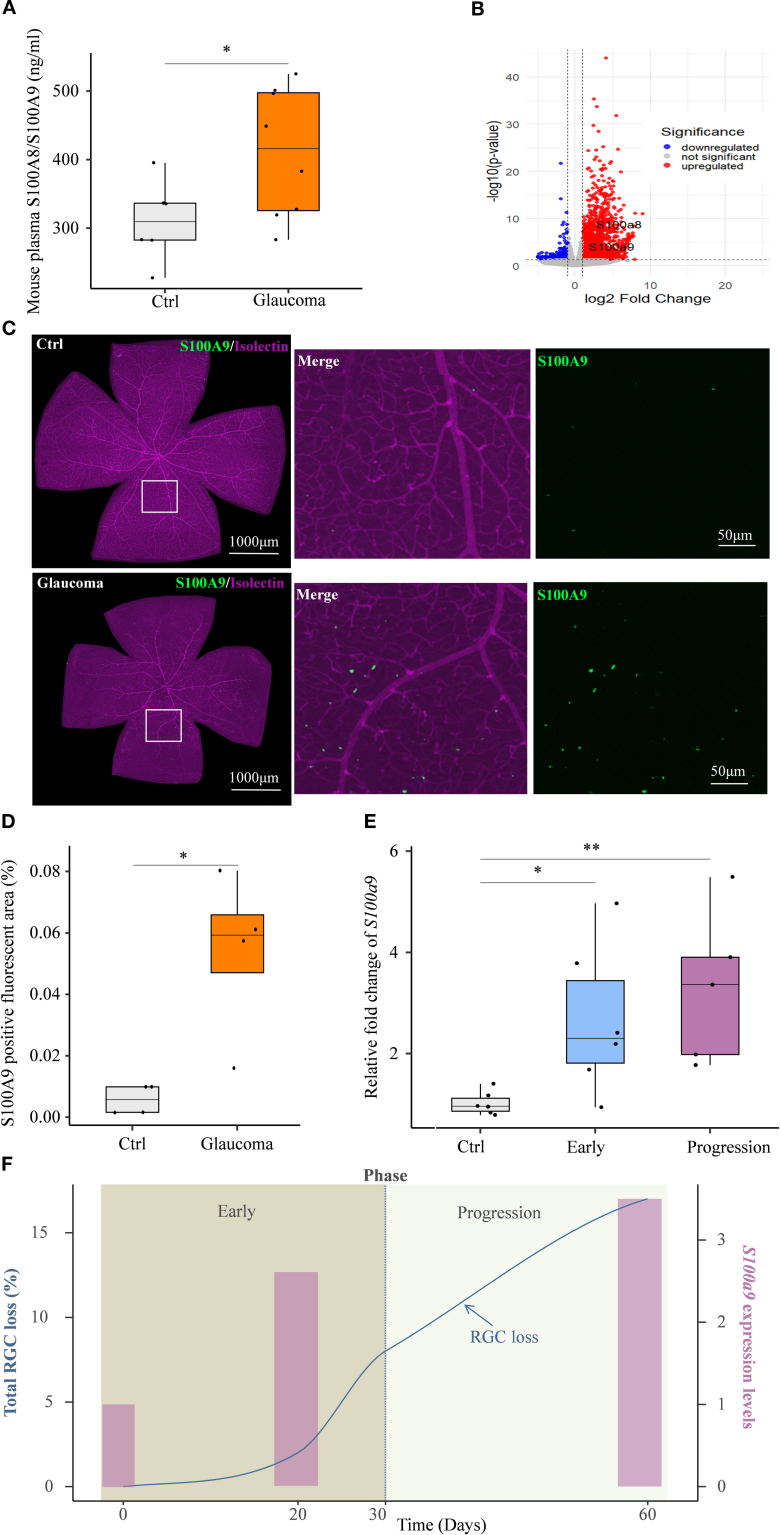
S100A9 association with progressive glaucomatous RGC degeneration in mice. **(A)** Elevated plasma S100A8/S100A9 levels in the EIOP glaucoma mice (n=8) compared to controls (n=6). **(B)** Volcano plot of GSE141725 (GEO dataset) showing elevated expression of *S100a8* and *S100a9* in glaucomatous retinal. **(C)** Representative confocal image of a retina flat mount co-stained with isolectin (purple) and S100A9 (green). **(D)** Quantification of the S100A9-positive fluorescent area (%) in control and EIOP-induced chronic glaucomatous retina (day 45) (n = 4 per group). **(E)** *S100a9* mRNA expression levels in EIOP-induced experimental glaucoma mice of Early phase (n = 6), and Progression phase (n = 5), compared with control mice (n = 6). **(F)** The kinetics of total RGC loss (blue solid line, derived from our previous study, left Y-axis) (12) and the *S100a9* mRNA expression levels (pink bars, representing the mean values from the control, early, and progressive phases in **Figure 3E**, right Y-axis) are presented across the time course. Statistical analyses were conducted using **(A)** a two-tailed unpaired Student’s t test, **(D)** the Mann–Whitney U test, and **(E)** the Kruskal–Wallis test followed by Dunn’s multiple comparisons test for multiple-group comparisons. *P < 0.05, **P < 0.01. Boxplots represent the median and interquartile range (IQR).

Circulating S100A8/S100A9 were also elevated in experimental glaucoma mice (**Figure 3A**). Transcriptomic analysis of the GSE141725 dataset revealed significant upregulation of *S100a8* and *S100a9* in glaucomatous retinal samples, with both genes exhibiting similar expression patterns (**Figure 3B**). Furthermore, immunofluorescence staining of retina flat mounts collected on day 45 revealed markedly increased S100A9 protein levels in glaucomatous retinas compared to controls (**Figures 3C, D**). Co-immunostaining with Isolectin B4, a vascular endothelial marker, showed that the majority of S100A9 signals were distributed perivascularly, external to the blood vessels (**Figure 3C**), indicating that the observed increase in S100A9 is not due to intravascular staining of circulating protein.

A previous study by our team showed that, in EIOP-induced glaucoma, RGC axonal injury was evident at day 20, whereas RGC loss progressively worsened from days 30 to 60 following microbead injection (12). Based on these observations, we investigated whether *S100a9* expression levels were associated with disease progression in EIOP-induced glaucoma mice. We found that retinal *S100a9* mRNA levels were markedly elevated prior to RGC loss in the early phase of glaucoma and further increased during the progressive phase (**Figure 3E**). *S100a9* expression increased in parallel with RGC loss during disease progression (**Figure 3F**). These results suggest that S100A9 expression levels are associated with glaucoma progression in mice.

### S100A9 contributes to RGC degeneration

**Figure 4:**
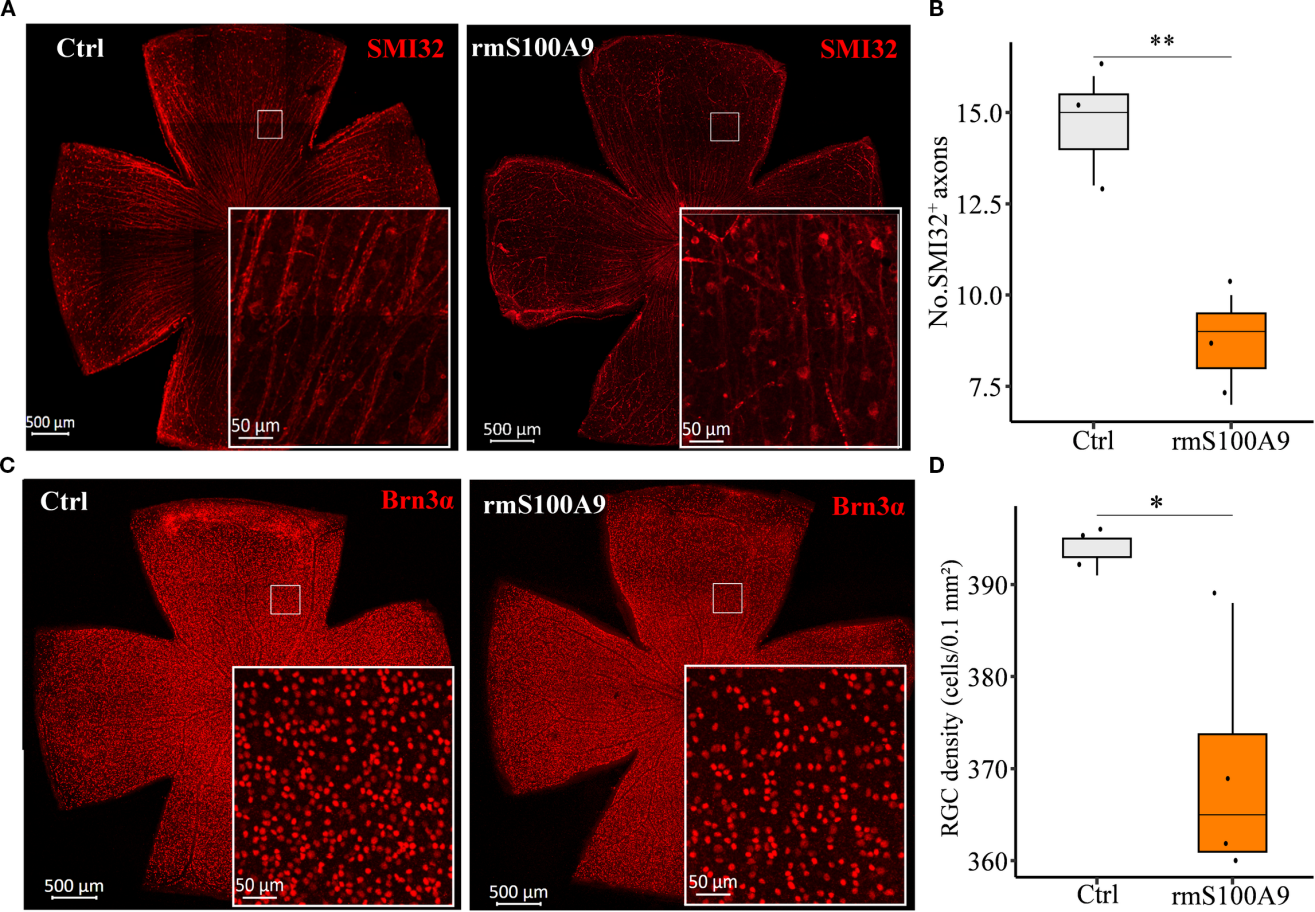
S100A9 induces glaucomatous-like retinal injury in mice. Representative confocal images of retina flat mounts immunostained for **(A)** SMI32 (red) and **(C)** Brn3α (red). **(B)** The number of SMI32+ RGC axons per microscopic field (size: 0.1 mm^2^) (n = 3). **(D)** Quantification of retinal ganglion cell (RGC) density (cells per 0.1 mm²) in control mice (n = 3 retinas) and mice intravitreally injected with recombinant murine S100A9 (rmS100A9) at day 50 (n = 4 retinas). Mann–Whitney U test; *P < 0.05, **P < 0.01. Boxplots represent the median and interquartile range (IQR).

To further investigate whether S100A9 contributes to RGC injury, rmS100A9 was intravitreally injected into healthy mice, and retinal flat mounts were collected on day 50. Immunofluorescence staining showed that SMI32, a marker of RGC axons, revealed disrupted and fragmented axons, along with a reduced number of SMI32^+^ fibers in rmS100A9-injected retinas (**Figures 4A, B**). Brn3α staining, a marker of RGC somas, demonstrated a significant reduction in RGC density compared to controls (**Figures 4C, D**).

### S100A9 might drive glia-mediated neuroinflammation leading to RGC degeneration

Previous studies have indicated that S100A9 contributes to CNS injury through neuroinflammatory mechanisms in neurodegenerative diseases. To determine whether S100A9 similarly contributes to RGC loss via neuroinflammatory pathways, we employed two complementary bioinformatics enrichment strategies using the GSE141725 dataset. First, we performed GO Biological Process (GO-BP) and KEGG pathway enrichment analyses on genes significantly correlated with *S100a9*, aiming to identify biological processes and pathways coordinately regulated with *S100a9*. Second, we conducted Gene Set Enrichment Analysis (GSEA) comparing groups with high versus low *S100a9* expression to determine functional pathways globally associated with elevated *S100a9* levels. We identified significant enrichment of inflammation and neuroinflammation terms within both the positively *S100a9*-correlated gene set (GO-BP analysis, **Supplementary Figure S1A**) and the GSEA comparing high versus low *S100a9* expression groups (**Supplementary Figure S1B**).

**Figure 5:**
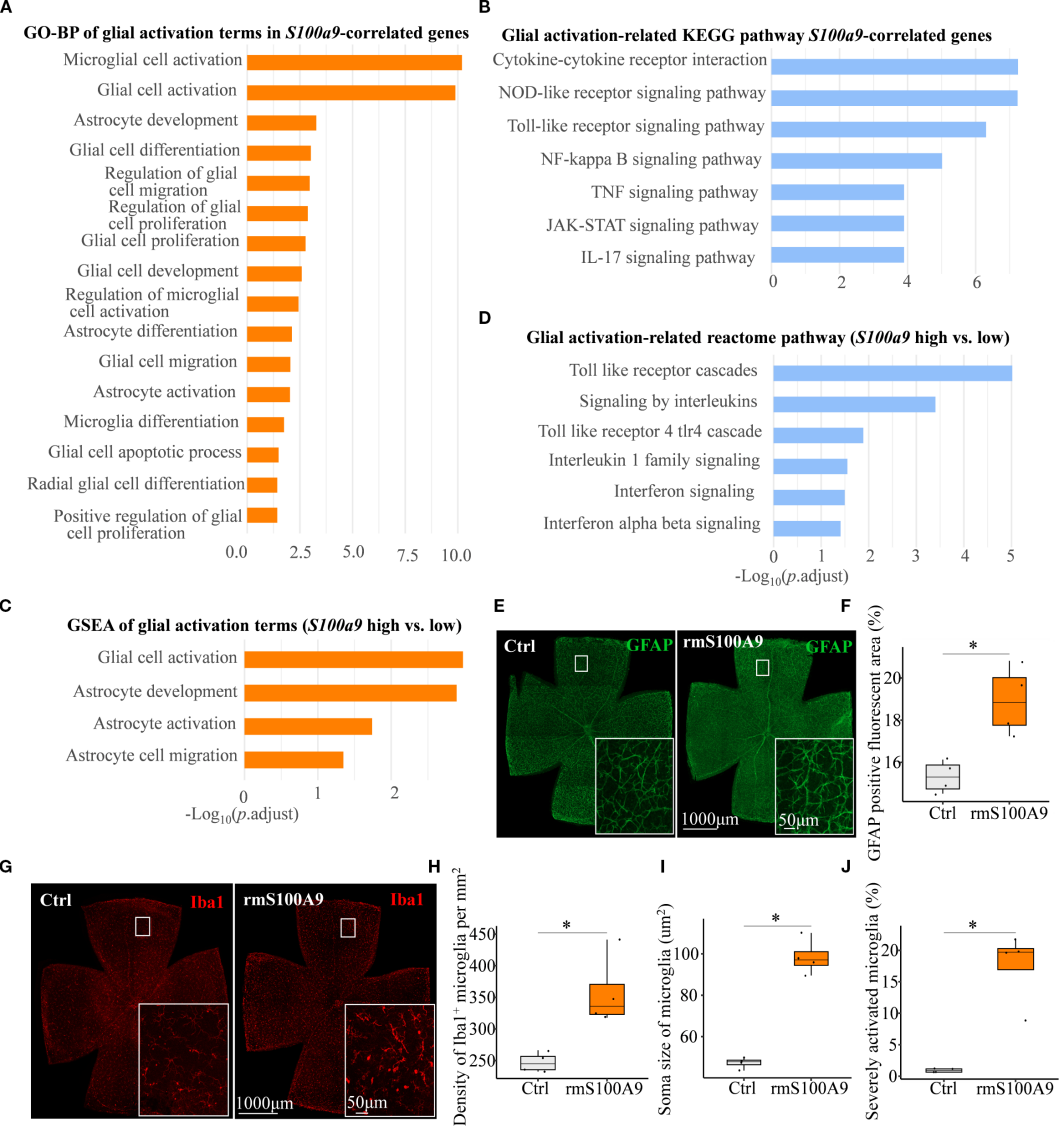
S100A9 induces glial activation. **(A)** GO-BP and **(B)** KEGG pathway enrichment analyses of glial activation–related terms and pathways among genes positively correlated with *S100a9* expression. **(C)** GSEA of GO-BP terms and **(D)** Reactome pathway enrichment comparing high vs. low *S100a9* expression in experimental glaucoma mice (GSE141725). Representative confocal images of retina flat mounts retinas immunostained for **(E)** astrocytes labeled with GFAP (green) and **(G)** microglia labeled with Iba1 (red) in control mice (n = 4 retinas) and mice intravitreally injected with rmS100A9 at day 21 (n = 4 retinas). **(F)** Quantification of GFAP-positive reactive area (%). **(H–J)** Quantification of Iba1-positive microglia, including cell density (cells/mm²), soma size, and the proportion of severely activated microglia. Quantification of glial cells was performed in four anatomically matched microscopic fields, individually selected from the superior, inferior, nasal, and temporal quadrants of each retinal flat-mount. Soma size was analyzed in at least 120 microglial cells per retina across these fields. Mann–Whitney U test; *P < 0.05. Boxplots represent the median and interquartile range (IQR).

As glial activation is a key component of neuroinflammation in neurodegenerative diseases, we next focused on S100A9-induced glial activation. We found that glial activation–related biological processes and pathways were significantly enriched in association with *S100a9* expression. These included GO biological process (GO-BP) and KEGG pathway enrichment of genes positively correlated with *S100a9* expression (**Figures 5A, B**), as well as GO-BP and Reactome pathway analyses based on gene set enrichment analysis (GSEA) comparing high versus low S100a9 expression groups (**Figures 5C, D**). Further experimental validation showed that, in mice at day 21 after intravitreal rmS100A9 injection, retinal flat mount staining revealed reactive astrocyte activation, characterized by hypertrophy and upregulated GFAP expression (**Figures 5E, F**). Additionally, Iba1 staining demonstrated excessive microglial activation, marked by soma enlargement and retraction of processes into an amoeboid-like morphology (**Figure 5G**). Quantitative analyses further confirmed a significant increase in microglial density (**Figure 5H**), enlarged soma area (**Figure 5I**), and a greater proportion of severely activated microglia was observed, characterized by markedly swollen soma, nearly complete retraction of processes, and, in some cases, an amoeboid morphology (**Figure 5J**). Interestingly, glial activation observed at day 20 in our previous study of EIOP-induced glaucoma mice was synchronized with that in the rmS100A9 model (11), also suggesting that increased S100A9 contributes to glial activation in glaucoma. These results support the hypothesis that S100A9-induced glial activation leads to retinal neuroinflammation, contributing to RGC degeneration.

**Figure 6:**
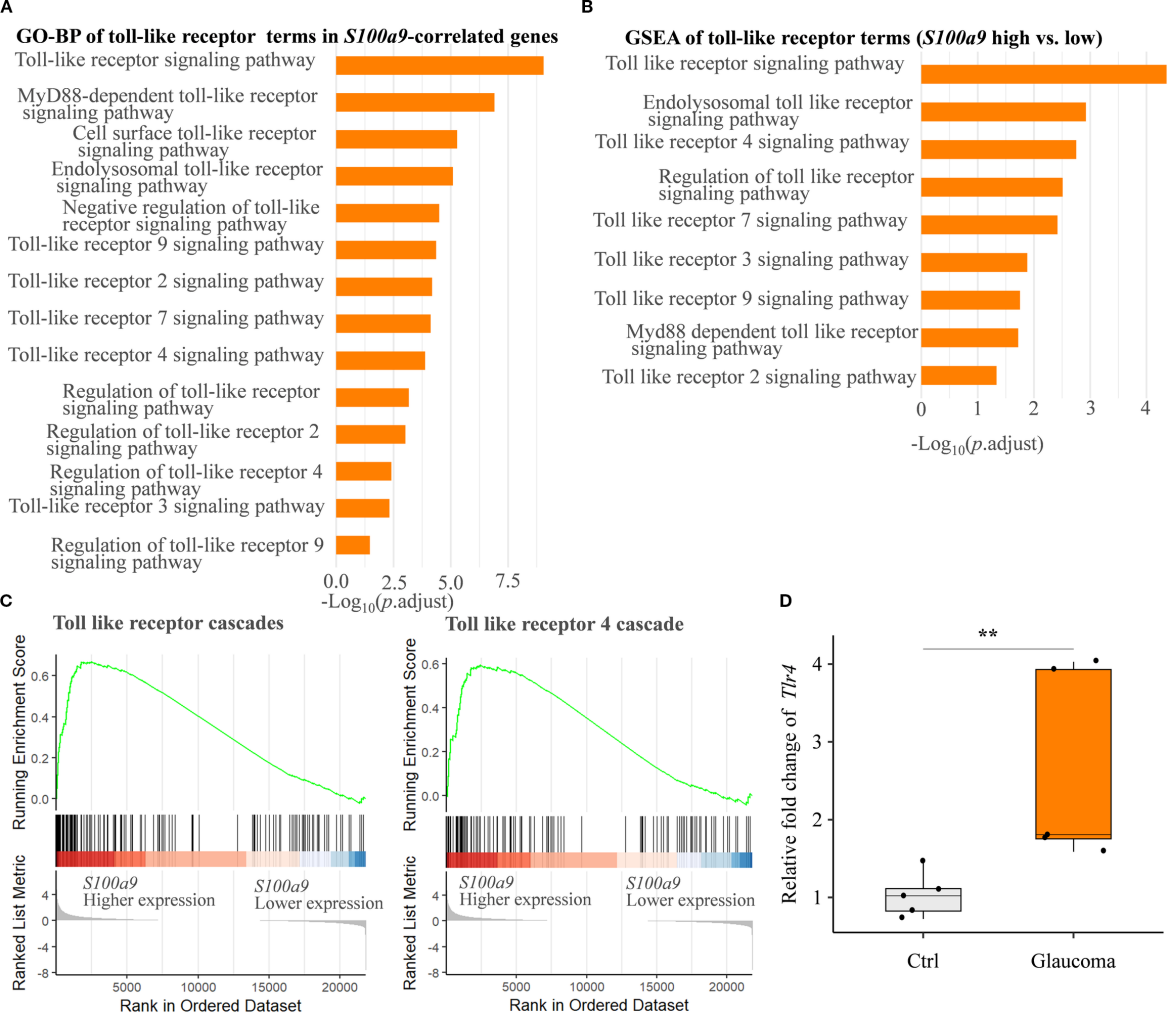
S100A9 activates the toll-like receptor signaling pathway. **(A)** GO biological process (GO-BP) enrichment and **(B)** GSEA of GO-BP terms related to toll-like receptor activation, based on genes correlated with positively *S100a9* expression and differential expression between *S100a9* high vs. low groups in experimental mice (GSE141725). **(C)** Reactome pathway enrichment plots showing enrichment scores for the toll-like receptor cascade and the toll-like receptor 4 (*Tlr4*) cascade, comparing *S100a9* high vs. low expression groups in experimental glaucoma mice (GSE141725). **(D)** Relative mRNA expression levels of *Tlr4* in EIOP-induced experimental glaucoma mice compared with control mice (n = 5 per group). Mann–Whitney U test; **P < 0.01. Boxplots represent the median and interquartile range (IQR).

Finally, to explore potential molecular mechanisms underlying glial activation induced by S100A9, we conducted a detailed pathway enrichment analysis. Our analyses revealed significant enrichment of the Toll-like receptor signaling pathway, including GO-BP (**Figures 6A, B**), KEGG (**Figure 5B**), and Reactome (**Figures 5D**, **6C**). Real-time qPCR further confirmed a significant upregulation of *Tlr4* mRNA in the EIOP-induced glaucoma mice (**Figure 6D**). Collectively, these results suggest that S100A9 induces glial activation through TLR4 signaling, contributing to RGC degeneration (**Supplementary Figure S2**).

## Discussion

S100A9 is an important pro-inflammatory mediator involved in neuroinflammatory injury to neuronal cells in CNS neurodegenerative diseases (17, 21). In this study, we found that S100A9 is involved in the development and stages of glaucoma, and we clarified the following points: (i) Circulating S100A9 levels are elevated in glaucoma patients and are positively correlated with disease severity. (ii) Our study revealed that S100A9 is associated with glaucomatous progression and promotes RGC loss in mice. (iii) S100A9 may contribute to RGC injury by activating glia-mediated neuroinflammatory responses. (iv) S100A9 may activate microglia and astrocytes through TLR4-mediated pathways. These results further support the role of neuroinflammation in glaucoma pathogenesis and highlight S100A9 as a key regulatory molecule driving glial overactivation in the glaucomatous retina.

S100A9 is a calcium-binding protein belonging to the S100 family. In the circulation, it is mainly derived from neutrophils and monocytes/macrophages (25). S100A8/S100A9, the stable form of S100A9, is widely used as a biomarker for assessing gut and systemic inflammation (24, 25). For example, fecal S100A8/S100A9 is the most commonly used non-invasive biomarker of intestinal inflammation in inflammatory bowel disease (IBD), facilitating diagnosis, monitoring disease progression, and evaluating therapeutic response (25, 26). S100A8/A9 is overexpressed during inflammation and has been implicated in the development and progression of CNS inflammatory diseases, including AD, PD, and multiple sclerosis (MS). In MS, serum S100A8/A9 concentrations are significantly elevated (27). In AD and PD, both fecal and circulating S100A8/A9 have been investigated as biomarkers of gut inflammation and disease activity/progression (18, 19). Recent studies have also shown that S100A9 protein levels in cerebrospinal fluid (CSF) may serve as a robust biomarker for early-stage AD diagnosis (28). The retina, as an extension of the CNS, shares common pathogenic mechanisms (29). Consistent with CNS neurodegeneration, our previous study demonstrated that fecal S100A8/S100A9 levels reflect intestinal inflammatory status in glaucoma (22). In the present study, we further demonstrated that circulating S100A9 levels were elevated in glaucoma patients and is positively correlated with disease stage. Notably, in our cohort, circulating S100A9 did not correlate with contemporaneous IOP. This is biologically plausible: glaucoma can progress despite effective or even normal IOP, indicating pressure-independent injury pathways (3). Consistently, ocular inflammatory mediators often show little cross-sectional correlation with IOP—an association further blurred by diurnal fluctuation, IOP-lowering therapy, and phenotypic heterogeneity—supporting S100A9 as an inflammatory readout partly orthogonal to IOP (30). We also confirmed that elevated S100A9 expression is associated with RGC loss in the retina of experimental glaucoma mice. Moreover, S100A9 upregulation occurred prior to RGC loss, and intravitreal injection of rmS100A9 led to significant RGC degeneration. Collectively, these results suggest that S100A9 contributes to RGC loss and could serve as a candidate biomarker for monitoring glaucoma.

Beyond its role as a biomarker, pro-inflammatory S100A9 also exerts direct pathogenic effects in the brain (17, 20, 21, 31). S100A9 plays a critical role in linking neuroinflammation with amyloid pathology through its intrinsic amyloidogenic properties and modulation of Aβ aggregation in AD (17). S100A9 knockout significantly reduced memory impairment and neuropathology in the Tg2576 AD mouse model by decreasing amyloid plaque formation (20). A recent study showed that persistent neuroinflammation promotes the spread of amyloidogenic S100A9 in brain tissue, potentially triggering the amyloid cascade involving α-synuclein and S100A9 (21), which contributes to PD pathogenesis—analogous to the co-aggregation of S100A9 and Aβ in AD. Additionally, amyloid-β is implicated in neurodegenerative disorders of both the brain and retina (32). Aβ has been reported to contribute to RGC apoptosis in glaucoma through caspase-3-mediated abnormal APP processing (33). Another study showed that Aβ colocalizes with apoptotic RGCs in experimental glaucoma, and targeting components of the Aβ formation and aggregation pathway effectively reduced glaucomatous RGC loss (34). Based on this, we speculate that S100A9 may facilitate Aβ aggregation, initiating an amyloid–neuroinflammatory cascade that contributes to RGC degeneration in glaucoma.

S100A9 has been shown to activate microglia and stimulate phagocytosis, resulting in synaptic and neuronal loss (35). Microglial activation is a central feature of neuroinflammation and serves as a key marker in current evaluations of neuroinflammatory states (36). Depending on the microenvironment, microglia can exert both protective and detrimental effects (6, 8). Excessive or chronic activation disrupts their homeostatic functions and contributes to irreversible neuronal damage (37, 38). Such pathological stimulation promotes the overproduction of pro-inflammatory cytokines, including TNF-α and interleukins, thereby exacerbating neuroinflammation and cytotoxicity, and leading to RGC degeneration (8). In this study, S100A9 expression remained consistently elevated from the early to the progressive phase in glaucoma mice and increased in parallel with RGC loss during disease progression. Notably, by day 21 after rmS100A9 injection, microglial activation was evident and accompanied by a markedly increased proportion of severely activated microglia, indicating a sustained and excessive microglial response. These results suggest that S100A9-driven aberrant microglial activation may exacerbate neuroinflammation and cytotoxicity, thereby contributing to glaucomatous neurodegeneration. Astrocytes also respond to pathological insults through reactive gliosis, a critical component of neuroinflammation (36). The term “reactive astrocytes” describes hypertrophic astrocytes with upregulated GFAP (39). In this study, we observed astrocytic hypertrophy and increased GFAP expression following S100A9 stimulation, indicating reactive gliosis. Collectively, these results suggest that S100A9 may contribute to RGC degeneration by inducing microglial and astrocyte activation–mediated neuroinflammatory responses.

Although glial activation has been extensively studied in glaucomatous retinas, the key regulatory signals driving excessive microglial activation in glaucoma remain incompletely elucidated (10). Evidence indicates that TLR expression is elevated in the retinas of glaucoma patients (9), and *in vitro* studies suggest that glaucomatous stress signals may activate glial immune responses via TLR pathways (4). In this study, we observed enrichment of the TLR signaling pathway and significant upregulation of *Tlr4* in experimental glaucoma models. Moreover, multiple studies have demonstrated that S100A9 activates microglia and astrocytes via the TLR4 signaling pathway in the CNS (40, 41). These results support the conclusion that S100A9 may promote microglial and astrocytic activation through TLR4 signaling in glaucomatous retinas.

Neuroinflammation leads to neurodegeneration primarily through the release of pro-inflammatory mediators, proteolytic enzymes, reactive oxygen species (ROS), and other cytotoxic factors by activated microglia and infiltrating immune cells (42). This pathological interaction forms a self-perpetuating cycle in which inflammation drives neurodegeneration, and neuronal injury, in turn, sustains the inflammatory response (36). Currently, no effective therapies are available to disrupt this vicious cycle (36). Given the strong interplay between inflammation and neurodegeneration, targeting neuroinflammation with blood–retina barrier (BRB) permeable agents may help slow disease progression. To achieve this, immunomodulatory therapies must effectively penetrate the BRB to ensure adequate delivery to affected neural tissues. A combined approach—suppressing immune activation while addressing the initiating insult—may offer greater therapeutic benefit. Despite disease- and tissue-specific variability in efficacy and safety—underscoring the context dependence of S100A9 inhibition—paquinimod has nonetheless shown benefit in several preclinical inflammatory and autoimmune models (including multiple sclerosis), notably reducing myeloid-cell infiltration into inflamed tissues and suggesting modulation of innate immunity (43). In this context, selective inhibition of S100A9 (a protein implicated in facilitating glial-mediated inflammation), pending definitive functional validation, could offer a potential avenue for rebalancing immune responses and mitigating neurodegeneration.

The limitations of our study include the following: (i) A larger number of glaucoma patients should be recruited, and multicenter, prospective cohort studies are needed to dynamically evaluate the role of S100A8/S100A9 in glaucoma. (ii) In this study, we performed intravitreal administration of recombinant S100A9, but were unable to conduct loss-of-function experiments. Future studies using S100A9 blockade or S100A9 knockout mice are necessary to further validate the role of S100A9 in glaucoma development and progression. (iii) The detailed pathogenic mechanisms by which S100A9 contributes to RGC injury require further investigation, including *in vivo* and *in vitro* co-culture studies.

In summary, our study suggests that S100A9 contributes to glaucomatous injury by promoting neuroinflammatory responses in retinal microglia and astrocytes through activation of the TLR4 pathway. It may also serve as a potential biomarker for assessing glaucoma stage. To the best of our knowledge, this is the first study to highlight this previously unexplored aspect. Functionally, by fostering a pro-inflammatory environment, S100A9 may contribute to chronic retinal inflammation and eventual RGC loss. While any therapeutic strategy aimed at retinal S100A9 remains speculative, our findings lay the groundwork for future mechanistic and interventional studies.

## Data Availability

The original contributions presented in the study are included in the article/**Supplementary Material**. Further inquiries can be directed to the corresponding authors.

## Supplementary material

Supplementary Table 1 Antibodies used for immunofluorescent staining.

Supplementary Figure 1 S100A9 induces neuroinflammatory response. **(A)** GO-BP enrichment analyses of neuroinflammatory –related terms among genes positively correlated with *S100a9* expression. **(B)** GSEA of GO-BP terms enrichment comparing high vs. low *S100a9* expression in experimental glaucoma mice (GSE141725).

Supplementary Figure 2 Schematic diagram of S100A9–TLR4–mediated glial activation leading to RGC degeneration.
