# Supplementary material for: Pro-inflammatory S100A9 contributes to retinal ganglion cell degeneration in glaucoma
Source: Front Immunol. 2025 Sep 25;16:1667097. doi: 10.3389/fimmu.2025.1667097 (PMC12507546; doi:10.3389/fimmu.2025.1667097)
Supplement: Supplementary Table 1 — Antibodies used for immunofluorescent staining. [file Table1.docx]

Supplement table 1

| Primary Antibodies | Source | Clone | Dilution Ratio |
| --- | --- | --- | --- |
| Brn3a | Abcam | EPR23257-285 | 1:500 |
| GFAP | Biolegend | 2E1.E9 | 1:400 |
| SMI32 | Biolegend | SMI32 | 1:500 |
| Iba1 | Wako | - | 1:200 |
| Isolectin GS-IB4 | Thermo Fisher Scientific | - | 1:200 |
| S100A9 | R&D | - | 1:150 |
| Second Antibodies | Source | Clone |  |
| Goat anti-Mouse IgG (H+L) Highly Cross-Adsorbed Secondary Antibody, Alexa Fluor Plus 488 | Thermo Fisher Scientific | _ | 1:500 |
| Goat anti-Mouse IgG (H+L) Highly Cross-Adsorbed Secondary Antibody, Alexa Fluor Plus 555 | Thermo Fisher Scientific | _ | 1:500 |
| Goat anti-Rabbit IgG (H+L) Highly Cross-Adsorbed Secondary Antibody, Alexa Fluor Plus 488 | Thermo Fisher Scientific | _ | 1:500 |
| Goat anti-Rabbit IgG (H+L) Highly Cross-Adsorbed Secondary Antibody, Alexa Fluor Plus 555 | Thermo Fisher Scientific | _ | 1:500 |
| Donkey anti-Goat IgG (H+L) Cross-Adsorbed Secondary Antibody, Alexa Fluor 488 | Thermo Fisher Scientific | _ | 1:500 |
