# Supplementary figures and images for: Pro-inflammatory S100A9 contributes to retinal ganglion cell degeneration in glaucoma
Source: Front Immunol. 2025 Sep 25;16:1667097. doi: 10.3389/fimmu.2025.1667097 (PMC12507546; doi:10.3389/fimmu.2025.1667097)

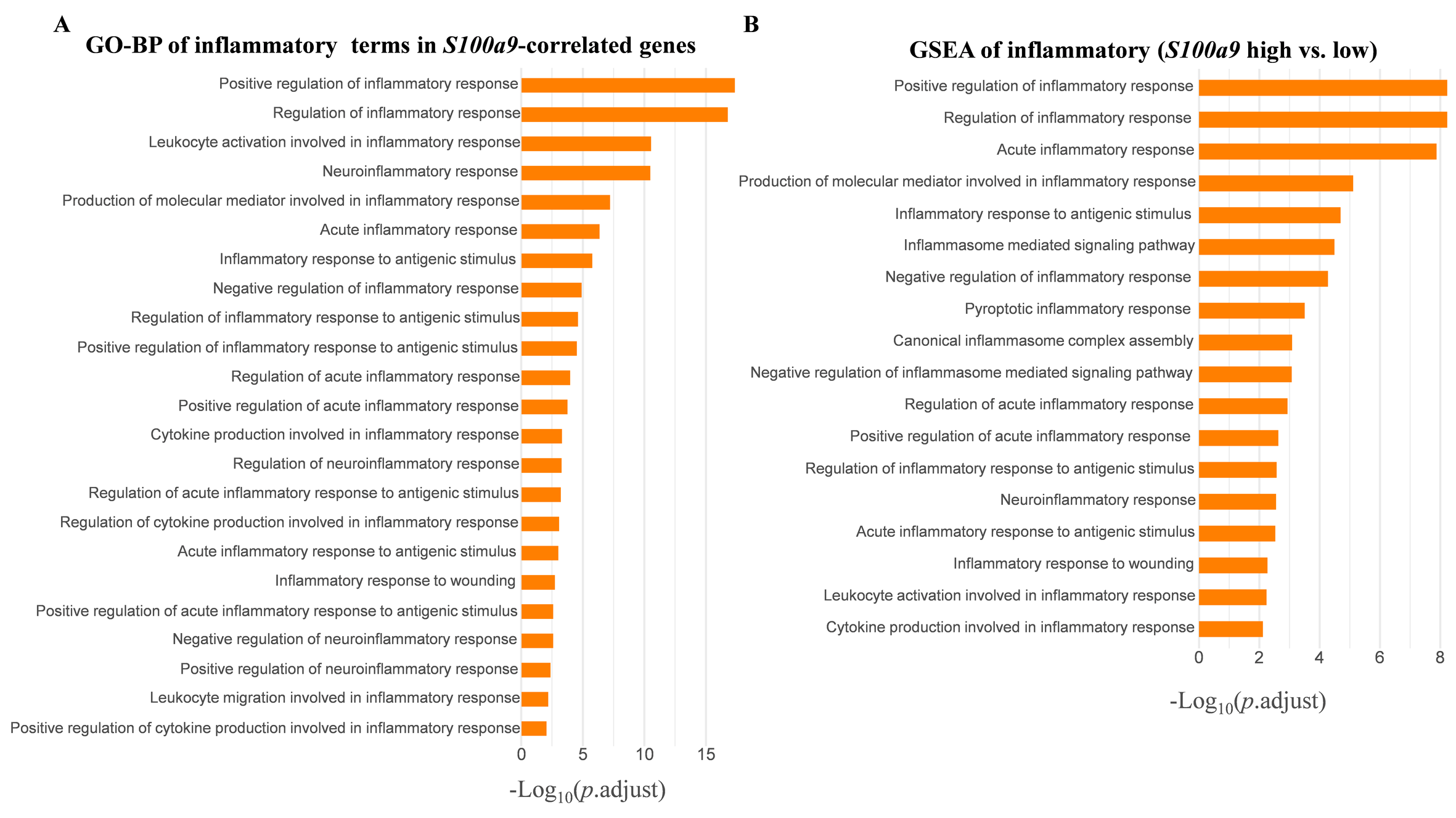

Supplement: Supplementary Figure 1 — S100A9 induces neuroinflammatory response. (A) GO-BP enrichment analyses of neuroinflammatory –related terms among genes positively correlated with S100a9 expression. (B) GSEA of GO-BP terms enrichment comparing high vs. low S100a9 expression in experimental glaucoma mice (GSE141725). [file Image1.tif]

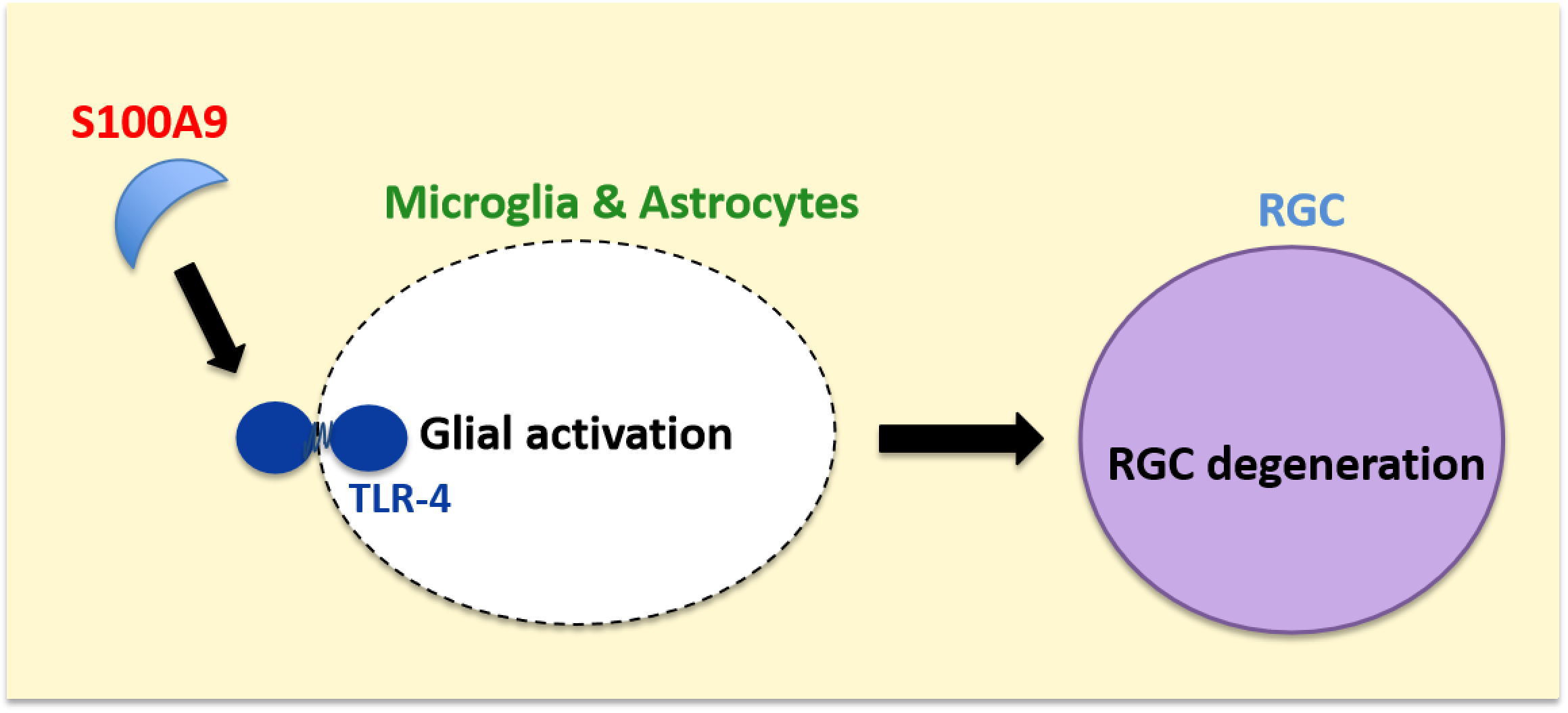

Supplement: Supplementary Figure 2 — Schematic diagram of S100A9–TLR4–mediated glial activation leading to RGC degeneration. [file Image2.tif]
